# Transcriptome Analysis of Epithelial and Stromal Contributions to Mammogenesis in Three Week Prepartum Cows

**DOI:** 10.1371/journal.pone.0022541

**Published:** 2011-07-29

**Authors:** Theresa Casey, Heather Dover, James Liesman, Lindsey DeVries, Matti Kiupel, Michael VandeHaar, Karen Plaut

**Affiliations:** 1 Department of Animal Science, Purdue University, West Lafayette, Indiana, United States of America; 2 Michigan State University, East Lansing, Michigan, United States of America; 3 Diagnostic Center for Population and Animal Health, Lansing, Michigan, United States of America; University of Pittsburgh, United States of America

## Abstract

Transcriptome analysis of bovine mammary development has provided insight into regulation of mammogenesis. However, previous studies primarily examined expression of epithelial and stromal tissues combined, and consequently did not account for tissue specific contribution to mammary development. Our objective was to identify differences in gene expression in epithelial and intralobular stromal compartments. Tissue was biopsied from non-lactating dairy cows 3 weeks prepartum, cut into explants and incubated for 2 hr with insulin and hydrocortisone. Epithelial and intralobular stromal tissues were isolated with laser capture microdissection. Global gene expression was measured with Bovine Affymetrix GeneChips, and data were preprocessed using RMA method. Moderated t-tests from gene-specific linear model analysis with cell type as a fixed effect showed more than 3,000 genes were differentially expressed between tissues (P<0.05; FDR<0.17). Analysis of epithelial and stromal transcriptomes using Database for Annotation, Visualization and Integrated Discovery (DAVID) and Ingenuity Pathways Analysis (IPA) showed that epithelial and stromal cells contributed distinct molecular signatures. Epithelial signatures were enriched with gene sets for protein synthesis, metabolism and secretion. Stromal signatures were enriched with genes that encoded molecules important to signaling, extracellular matrix composition and remodeling. Transcriptome differences also showed evidence for paracrine interactions between tissues in stimulation of IGF1 signaling pathway, stromal reaction, angiogenesis, neurogenesis, and immune response. Molecular signatures point to the dynamic role the stroma plays in prepartum mammogenesis and highlight the importance of examining the roles of cell types within the mammary gland when targeting therapies and studying mechanisms that affect milk production.

## Introduction

The bovine mammary gland undergoes morphological changes throughout the reproductive cycle. During the prepubertal/pubertal stages the gland undergoes a period of allometric growth and development characterized by the branching expansion of epithelial parenchyma and associated stroma into the fat pad. Once expansion is complete, growth and development of the gland enters a steady state period until the onset of pregnancy when growth, development and differentiation (i.e. mammogenesis) are reinitiated. Mammogenesis continues throughout the first pregnancy as epithelial structures mature and prepare for lactation. At the onset of lactation epithelial cells begin to secrete milk. Milk synthesis and secretion continue until milk removal ceases.

At cessation of milk removal, or dry-off, the gland undergoes a period of rapid involution. One week following dry off there is an increase in apoptosis in alveolar structures [1], a decrease in luminal content and an increase in the area occupied by stroma [2], [3], [4], [5], [6]. If the animal is pregnant, the period of involution is followed by mammogenesis as the mammary gland prepares for another round of lactation. The interval between successive lactations is referred to as the dry period. A dry period of 55±5 days is typically used in dairy operations to maximize milk yield in the subsequent lactation [7], [8]. Mammogenesis during the dry period begins 3–4 wk prior to parturition and is characterized by redevelopment of the alveoli and increased collagen synthesis. These changes indicate that the bovine mammary gland is remodeled through changes in both its cellular and extracellular content during the dry period [4], which result in an increase in luminal area and decrease in stromal area through 1 week prepartum [2], [3], [4], [5], [6].

At the systemic level mammary growth and development are regulated by hormones. Hormones either act directly to influence growth and differentiation of mammary epithelial cells or indirectly by influencing production of local signaling factors. Steroid hormone induced mammary development often occurs through indirect mechanisms and requires stromal cooperation for induction of epithelial proliferation and morphogenesis [9], [10]. Stromal cells influence epithelial cell behavior by secreting growth factors and/or by altering the composition of the ECM in which epithelial cells reside. The ECM provides structural support for cells, acts as a reservoir for signaling molecules, and plays an active role in initiating cell signaling. Composition and architecture of both the interstitial stromal ECM and the basement membrane ECM change throughout mammary development and in response to systemic and local factors [10], [11], [12], [13], [14]. Remodeling of the ECM by enzymes such as the metalloproteainases (MMP) removes barriers for epithelial growth and releases signaling molecules from stromal reservoirs [11]. The mammary gland of dry cows at three weeks prepartum has been characterized as being in a period of epithelial cell redevelopment with a high rate of ECM synthesis [2], [3], [4], [5]. ECM synthesis is needed to prepare the gland for lactation, as two converging signals are required to turn on milk protein gene expression in mammary epithelial cells, one from lactogenic hormones and the other from the ECM [15], [16], [17].

Studies conducted to characterize transcriptome changes that occur during mammary development have provided insight into key processes involved in signaling and morphogenesis of the mammary gland (for example [18], [19], [20]). However, these studies and others have only examined global gene expression of combined stromal and epithelial cells, and consequently do not account for individual contribution of the cell types to mammary development. The objective of this study was to identify key differences in gene expression and gene signaling pathways in the epithelial and intralobular stromal compartments. Laser capture microdissection was used to isolate populations of epithelial and intralobular stromal cells from mammary tissue of dry cows three weeks prepartum. Analysis of epithelial and stromal global gene expression revealed the distinct contribution of each population to prepartum mammogenic transcriptomes of dry cows and provided insight into how these tissues interact during development.

## Materials and Methods

### Animals and tissue collection and processing

All animal handling and surgical procedures were performed humanely under the approval of the Michigan State University's Institutional Animal Care and Use Committee (approval AUF # 08/05-120-00). Six multiparous Holstein cows from the Michigan State University Dairy Cattle Teaching and Research herd were impregnated between 90 and 105 after the onset of lactation. Cows were dried off after being milked for 310±12 days and had an average dry period length of 55±5 days. A mammary biopsy was obtained three weeks before expected calving date, during the window of expected mammogenesis [2], [3], [4], [5], using the method described by Farr et al. (1996) with several modifications. Cows were restrained and sedated with an intramuscular injection of xylazine hydrochloride (35–45 µg/kg of body weight, Rompun; Bayer Animal Health, Shawnee, KS). The biopsy site was shaved, washed and scrubbed with iodine and 70% ethanol. Topical lidocaine gel was applied to the biopsy site and 3 ml lidocaine injection was injected s.c. into the mammary gland to improve cow comfort. Approximately 1 g of tissue was collected using a stainless steel, retractable biopsy tool (AgResearch, NZ). Sterile absorbable gelatin sponge (Gelfoam; Pfizer, NY, NY) was placed in the biopsy site when excessive bleeding occurred. The incision area was closed with surgical staples and Autoclips (Becton, Dickinson and Company, NJ). This study was part of a larger study that describes biopsy and effect of biopsy on subsequent production in greater detail (Figure S1 [6]), including post-hoc analysis that showed biopsies were taken 19±2 days before expected calving, which corresponded to 259±1 days gestation.

Biopsied tissue was immediately placed in cold media (Waymouth's Media 752/1; Sigma, St Louis, MO; with 50 µg gentamicin per ml, Invitrogen, Carlsbad, CA; and 100 U penicillin – 100 µg streptomycin per ml, Sigma) and transported to the lab. Biopsied tissue was washed three times in phosphate-buffered-saline (PBS) containing; 100 U penicillin – 100 µg streptomycin per ml and 1.5 µg Amphotericin-B per ml (Invitrogen) and cut into 0.5 cm^3^ explants. Explants were placed on siliconized lens paper, and cultured in a humidified chamber (5% CO_2_, 50% O_2_) at 37°C for 2 h in Waymouth's media supplemented with 5 µg/ml insulin (Sigma), 0.1 µg/ml hydrocortisone (Sigma) and 50 µM 5-bromo 2-deoxyuridine (BrdU; Sigma) to measure rate of proliferation. Following explant culture, tissue was embedded in Tissue Tek OCT cryostat embedding compound (Ames Co, Division of Miles Laboratory, Elkhart, ID), flash frozen in liquid nitrogen and stored at −80°C, or placed in 10% buffered formalin for 24 hr at RT. Tissues embedded in OCT were used for laser capture microdissection. Tissues fixed in buffered formalin were embedded in paraffin and used to measure rate of BrdU incorporation or to measure protein expression with immunohistochemistry.

### Detection of rate of BrdU incorporation

Paraffin-embedded formalin fixed tissue was cut into 5 µm sections and placed on charged slides. The Zymed BrdU Staining Kit (Invitrogen) was used to detect BrdU incorporation into cells. Stained slides were examined with a Nikon Eclipse 50i light microscope (Nikon Instruments Inc., Melville, NY). Five photomicrographs were taken per slide at 200X magnification and images were captured at a size of 2560 by 960 pixels with 32-bit per pixel depth and saved in tagged image file format (TIFF) in Image Pro Plus 5.1 (Media Cybernetics, Bethesda, MD). Four classes of cells were counted (proliferating epithelial, non-proliferating epithelial, proliferating stromal and non-proliferating stroma) using Image Pro Plus software by manually tagging stained and unstained epithelial and stromal cells in the section. Approximately 3,300 epithelial or stromal cells were counted/cow to determine percent BrdU labeled index. Data were expressed as mean percent proliferating ± standard error of the mean, and a paired student t-test was used to determine difference.

### Laser capture microdissection (LCM) and isolation of total RNA

Serial tissue sections (7 µm) were prepared from OCT preserved tissue with a cryostat microtome using RNase-free techniques and stored at −80°C until use (less than 8 weeks). On day of LCM, tissue sections were fixed and stained in In Situ Hybridization Pap Jars (Evergreen Scientific, Los Angeles, CA) using the Histogene Kit (Molecular Devices, Sunnyvale, CA) following manufacturer's directions and left in xylene until LCM to ensure tissue was dehydrated.

**Figure 1:**
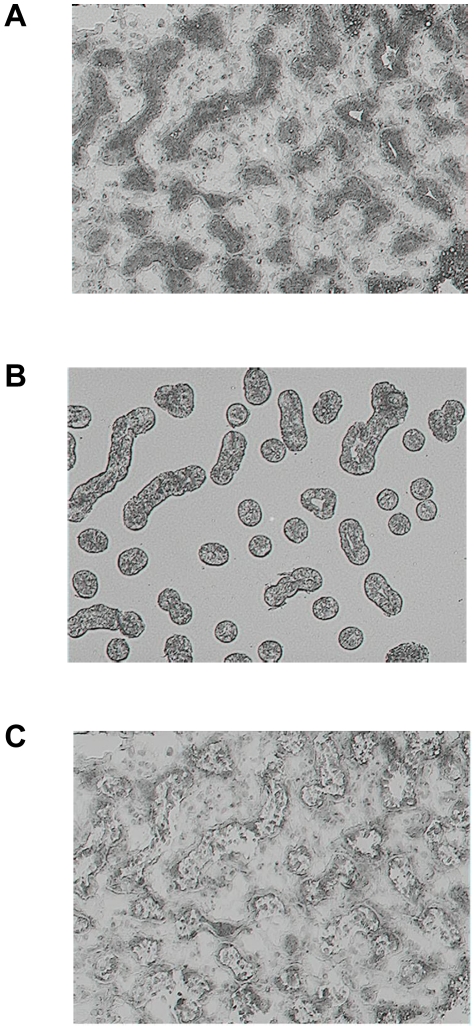
Images of the mammary tissue captured to document cell types captured with laser capture microdissection. Images were captured using light microscopy at 200X A) prior to laser capture microdissection (LCM) B) following LCM and of the C) cap to document populations of epithelial cells isolated from slide.

Populations of epithelial cells and intralobular stromal tissue were isolated by LCM using an Arcturus PixCell IIe LCM system (Arcturus Engineering now Molecular Devices) with CapSure LCM Transfer Film (Molecular Devices) according to manufacturer's protocol. Although intralobular stromal tissue contains a mixed population of cells that include fibroblasts, endothelial, nervous and immune cells, our goal was to enrich stromal captures with fibroblasts so during LCM care was taken to avoid blood vessels and obvious immune cells. Pictures were taken of histological sections before and after LCM to document cell types counted and tissue captured (Figure 1).

Tissue captured onto transfer film was immediately immersed in 50 µl of isolation buffer from Acturus' Picopure kit (Molecular Devices). To obtain a minimum of 20 ng of total RNA, lysates were pooled from two slides of the same sample for epithelial captures and four slides from stromal captures. Total RNA was isolated following manufacturer's protocol for laser captured frozen tissue. Recovered RNA was analyzed using a Bioanalyzer 2100 (Agilent Inc; Palo Alto, CA) to determine quality and quantity by use of the Picochip and multiple standards. RNA integrity number (RIN) of tissue off the slides was >8.0 for all samples.

### Target preparation and microarray analysis

NuGen WT Ovation Pico Technology, a high-amplification method for small amounts of RNA, was used for Target preparation following manufacturer protocol to generate products for the Bovine Affymetrix GeneChip. Gene chips were hybridized and scanned using standard Affymetrix protocols. Briefly, gene chips were hybridized in an Affymetrix 640 Hybridization oven at 45°C for 16 hours with 60 rpm rotation. After hybridization, gene chips were washed according to Affymetrix protocol on a Fluidics station using non-stringent (6xSSPE, 0.01% Tween 20, 0.005% antifoam) and stringent (100 mM MES, 0.1M NaCl, 0.01% Tween20) wash buffers. Arrays were stained using SAPE stain and antibody solutions. SAPE contains 2 ug/ul BSA, 10 ug/ml Streptavidin Phycoerythrin (SAPE) in 100 mM MES, 1M NaCl, 0.05% Tween 20 and 0.005% antifoam. The antibody solution contains: 2 mg/ml BSA, 0.1 mg/ml goat IgG, 3 ug/ml biotinylated anti-streptavidin antibodies in 100 mM MES, 1M NaCl, 0.05% Tween 20 and 0.005% antifoam. Genechips were then scanned using an Affymetrix Genearray scanner GSC3000, with 7G upgrade. The efficiency of amplification and hybridization were assessed by incorporating Affymetrix Poly-A RNA and hybridization controls with every sample.

GeneChip quality was evaluated by examining the 3′ to 5′ ratio of the GAPDH housekeeping gene, as the 3′ to 5′ ratio gives an indication of the integrity of the starting RNA (Affymetrix Support; www.affymetrix.com/support). We found that epithelial and stromal samples exhibited excellent overall signals with GAPDH 3′ to 5′ ratio of <1.5, which suggests that starting material was of suitable quality for measuring gene expression [21]. Scaling factor among all GeneChips was less than 3-fold, and thus suitable for comparison of differential expression (Affymetrix Support). All data are MIAME compliant and have been deposited in the Gene Expression Omnibus (GEO; www.ncbi.nlm.nih.gov/geo, accession no. GSE18768).

Gene expression analysis was performed using BioConductor version 2.0 software [22]. Data preprocessing was performed using the RMA (Robust Multiarray Analysis) method, which includes a quantile normalization step, across 24 arrays as implemented in the BioConductor package [22], [23], [24]. Twelve of the twenty-four arrays were the focus of this paper, and were thus generated with total RNA isolated from epithelial (n = 6) and stromal (n = 6) tissues captured from mammary explants cultured as described (Figure S1). Differential expression analysis was tested using moderated t-tests from gene-specific linear model analysis with cell type as a fixed effect and implemented in the MAANOVA (microarray analysis of variance) package of R [25]–[26]. Probes that were not expressed (P>0.05; Affymetrix MAS5.0) in at least 4 out of 6 epithelial and stromal arrays (i.e. at least 8 arrays total) were deleted, which accounted for 58% of the genes on the array. Thus we analyzed expression of 10,031 out of 24,016 genes (42%). Further, to control for multiple testing, corresponding nominal p-values were adjusted using the false discovery rate method of Bejamini and Hochberg [27].

The Functional Annotation Tool available through the Database for Annotation, Visualization and Integrated Discovery (DAVID) 2008 (http://david.abcc.ncifcrf.gov/; [28], [29]) was used to identify and analyze gene sets significantly enriched with genes differentially expressed between epithelial and stromal tissue at nominal P<0.05. Ingenuity Pathways Analysis (IPA) program (Ingenuity Systems, Mountain View, CA) was used to identify the biological functions, canonical pathways and functional networks enriched with genes unique to epithelial or stromal transcriptomes. The bovine Affymetrix array knowledge base was selected when data were uploaded into IPA, and filters for data analyses were set at P<0.05. Gene functions were determined using the NCBI Entrez Gene database [30].

After uploading the data into IPA, genes expressed at a greater level in epithelial and stromal transcriptomes were overlaid onto a global molecular network developed from information contained in the Ingenuity Pathways Knowledge Base. A description of the IPA networks methods can be found at www.ingenuity.com/products/appnotes. Graphical representation networks are restricted to a total of 35 genes/network. A “score” is assigned to each of the generated networks and is calculated by dividing the number of focus genes in the network by the total genes in the network. In addition, statistical probability (Fischer's exact test) of finding this ratio by chance (P-value) is also provided. This is displayed as the negative log of that P-value. Therefore, networks with scores of 2 (P<0.01) or higher have at least a 99% confidence of not being generated by random chance alone.

Canonical pathways analysis was used to identify pathways from IPA library that were most significant to the input data set. The significance of association between the data set and the IPA curated canonical pathways is measured in two ways: 1) A ratio is calculated of the number of genes from the data set that map to the pathway divided by the total number of genes in the archived canonical pathway, and 2) Fischer's exact test is used to calculate a P-value to determine the significance of the association between the genes in the dataset and the canonical pathways.

### Quantitative polymerase chain reaction (qPCR)

Total RNA (640 pg) was reverse transcribed into cDNA, ligated and amplified for 8 hrs using Qiagen Quantitect Whole Transcriptome Kit following manufacturer's instructions. cDNA was used for qPCR. The qPCR analysis was performed using the StepOnePlus™ Real-Time PCR Systems (Applied Biosystems, Foster City, CA), ABI TaqMan Gene Expression Master Mix and unique TaqMan® Gene Expression Assays (Applied Biosystems) specific for bovine: FN1 (fibronectin; Bt00415008_m1); IGF1 (Bt03252280_m1); NIDI (nidogen 1/entactin Bt03272040_m1); CSN2 (beta-casein; Bt03217428_m1); CDH1 (cadherin 1, type 1, E-cadherin; Bt03210091_m1); PRLR (prolactin receptor; Bt03207221_m1), and RP18S (Bt03225196_g1) as the reference gene. The mean sample threshold cycle (CT) and mean of the reference CT for each sample were calculated from duplicate wells. The relative amounts of target gene expression for each sample were then calculated using the formula 2^−ΔΔCT^ [31].

### Immunohistochemistry (IHC)

Immunohistochemistry was used to visualize in situ expression of MMP3 and fibronectin proteins in epithelial and stromal tissue in 5 µm sections cut from paraffin embedded sections. Prior to immunohistochemistry, slides were incubated overnight at 60°C, deparaffinized in xylene, rehydrated through a graded series of ethanol washes (100%, 95%, 70%, and 60%) and rinsed in PBS. Endogenous peroxidases were blocked for 15 minutes in 3% hydrogen peroxide in methanol. Sodium citrate antigen-retrieval was performed in a 100°C water bath. Sections were then incubated for 10 min at RT in non-immune blocking serum block (Histostain kit; Zymed Laboratories, San Francisco, CA), excess blocking serum was blotted off and sections were incubated with the primary mouse monoclonal anti-fibronectin (50 µg/ml; 1 hr; MS-1351-R7; Lab Vision Products, Fremont, CA) or mouse polyclonal anti-MMP-3 (1∶200; 45 min; 3523R-100; Biovision Inc., Mountain View, CA) at RT diluted in blocking solution. Sections were washed in PBS and incubated with a broad-spectrum secondary antibody conjugated with streptavidin-peroxidase (Histostain kit; Zymed Laboratories Inc.), followed by a PBS wash and 3 min incubation with 3′, 3′-diaminobenzidine chromogen substrate. All slides were counterstained with hematoxylin, dehydrated in a graded series of alcohols, cleared in xylene, and mounted with Histomount (Zymed).

## Results

The relative rate of epithelial (0.34%±0.26) and stromal (0.37%±0.17) cell proliferation was not different in mammary tissue from dry cows 3 weeks prepartum. Analysis of frequency of differentially expressed genes by P-value (Figure S2) revealed that epithelial and stromal transcriptomes were significantly different. Gene sets were searched for expression of epithelial-specific and stromal fibroblast-specific markers to confirm enrichment of cell populations by LCM. Ductal epithelial cytokeratin KRT7 and simple epithelial cytokeratin KRT8 were expressed 15.1-fold (P = 4.95 E-6; FDR = 0) and 8.1- fold (P = 9.89 E-6; FDR = 0) greater in the epithelial gene set, respectively, and the fibroblast cell specific markers VIM (vimentin) and S100A4 (fibroblast specific protein 1) [32] were expressed 11.3 fold greater (P = 7.39 E-7; FDR = 0) and 18.8-fold greater (P = 2.16 E-6; FDR = 0) in the stromal fibroblast gene set.

### Functional annotation clusters of genes expressed at a greater level in epithelial or intralobular stromal compartments

More than 3,000 genes (P<0.05) were differentially expressed between epithelial and stromal cell populations (Table 1). Functional Annotation Clustering in DAVID showed that genes that were expressed at a greater level in epithelial tissue were clustered into 131 functional groups; seven of these groups had a median significance of P<0.0001 (enrichment score (ES) >4; Supplemental Information S1, worksheet A). Analysis of these seven functional groups revealed that the epithelial transcriptome was enriched for genes with intracellular functions including regulation of metabolism and protein synthesis (Table 2). Genes that were expressed at a greater level in stromal tissue were clustered into 90 functional groups; five of these groups had a median significance of P<0.0001 (ES>4; Supplemental Information S1, worksheet B). Analysis of these five clusters revealed the stromal fibroblasts were enriched with transcripts associated with extracellular proteins and processes associated with adhesion, development and wound healing (Table 2; Supplemental Information S1, worksheet B).

**Table 1:** Summary of number of genes differentially expressed at P-value cut-off and associated maximal FDR.

	Higher in stroma		Higher in epithelium	
P-value	no.	max. FDR	no.	max. FDR
0.05	1108	0.16	1993	0.16
0.01	761	0.05	1016	0.05
0.001	487	0.01	394	0.01

**Table 2:** Representative gene sets within the functional groups significantly enriched (P<0.0001) with genes expressed at a greater level in epithelial or stromal tissue and identified using Functional Annotation Clustering in NIH DAVID.

Gene set	Score*	No.
Epithelial		
GOTERM_CC_ALL:GO:0005737∼cytoplasm	28.37	332
GOTERM_BP_ALL:GO:0009058∼biosynthetic process	20.23	140
GOTERM_BP_ALL:GO:0044267∼cellular protein metabolic process	14.17	189
GOTERM_CC_ALL:GO:0031090∼organelle membrane	10.91	74
GOTERM_CC_ALL:GO:0005739∼mitochondrion	7.95	83
GOTERM_CC_ALL:GO:0031410∼cytoplasmic vesicle	5.07	28
GOTERM_CC_ALL:GO:0033279∼ribosomal subunit	4.65	16
Stromal		
GOTERM_CC_ALL:GO:0031012∼extracellular matrix	12.27	28
GOTERM_BP_ALL:GO:0007155∼cell adhesion	10.52	32
SP_PIR_KEYWORDS:signal	7.96	93
GOTERM_BP_ALL:GO:0007275∼multicellular organismal development	4.86	44
GOTERM_BP_ALL:GO:0009611∼response to wounding	4.56	22
GOTERM_BP_ALL:GO:0009611∼response to wounding	4.56	22

*Score is DAVID's enrichment score, the –log of the median P-value for the functional group.

**Figure 2:**
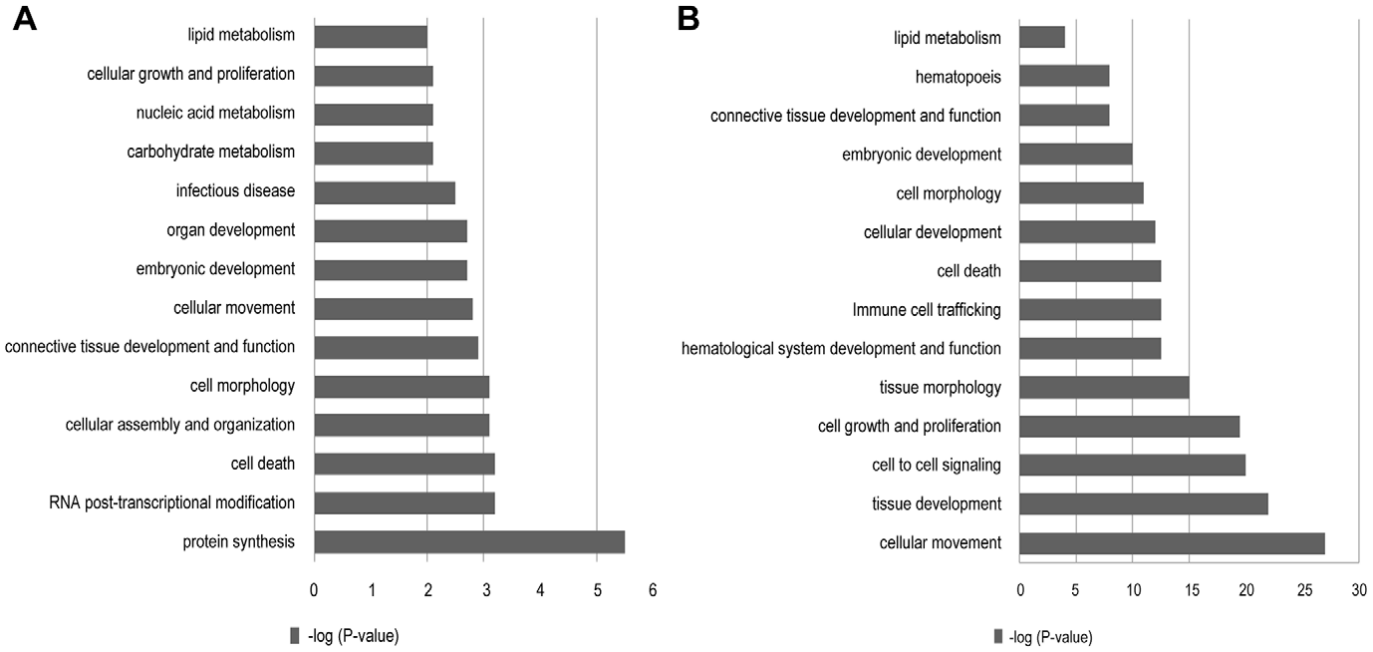
Molecular and cellular function ontologies enriched with genes expressed at greater level in epithelial and stromal tissues. Ontologies more highly enriched by genes expressed at greater level in A) epithelial and B) stromal tissue in mammary glands of three week prepartum dry cows generated using IPA. Y-axis = −log (P-value).

Enrichment of molecular and cellular functions (P<0.005) of IPA generated gene sets were also examined. For epithelial tissue, protein synthesis (86 genes; P<0.0001) was the most highly enriched gene set (Figure 2A); other notable sets included cellular assembly and organization (48 genes; P = 0.0004), connective tissue development and function (21 genes; P = 0.001), and reproductive function and development (17 genes; P = 0.002). Cellular movement (170 genes; P<0.0001) was the most highly enriched stromal compartment gene set (Figure 2B). Five molecular and cellular functional ontologies (P<0.05) were redundant between epithelial and stromal transcriptomes and included cellular growth and proliferation, cell-cell signaling and interaction, lipid and carbohydrate metabolism, and cell death (Figure 2).

**Figure 3:**
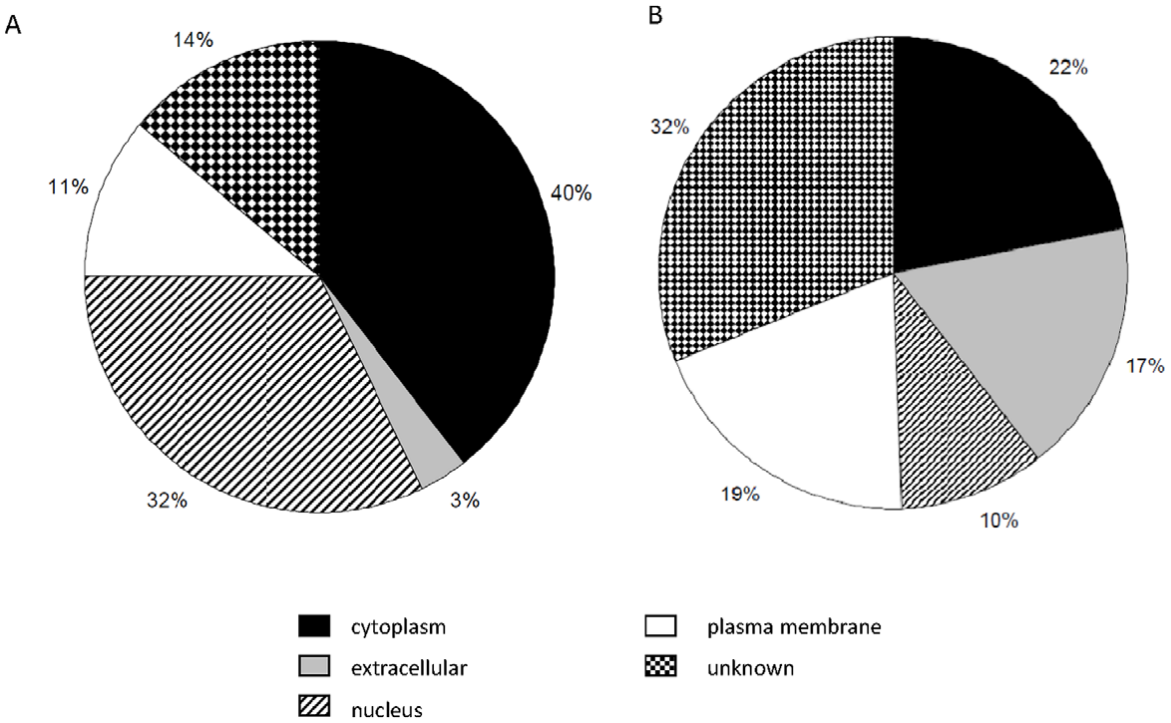
Cellular distribution of gene products expressed in epithelial and stroaml tissue. Genes expressed at a greater level in A) epithelium and B) intralobular stroma of dry cows three weeks prepartum.

The cellular distribution of proteins encoded by genes expressed at a greater level in mammary epithelium or stroma was investigated using IPA *Molecules* tab and sorting genes by location. The majority of genes expressed at a greater level in the epithelium encoded proteins that function within the cytoplasm and nucleus (Figure 3). In contrast many genes expressed at a greater level in intralobular stroma encoded proteins that function in the plasma membrane and extracellular space (Figure 3).

### Network analysis of genes expressed at a greater level in epithelial or intralobular stromal cells

Functional analysis of the genes expressed at a greater level in epithelial or stromal tissue identified the biological functions of the top five IPA generated networks (Table 3; ure S3A-E and Figure S4A-E). The epithelial network with the highest score (network 1, score 49) was characterized by genes that encoded cytoskeletal proteins (ACTA1, ACTG1) and proteins that regulate transcription (FOS, BARX2, GTF21RD1, MLL3, NACA, NCOA6, PFDN1) and translation, including ten ribosomal proteins (Figure S3A; Supplemental Information S1, worksheet C). Epithelial network 2 (score 46) was characterized by a MAPK node and genes encoding proteins that function in the nucleus to process RNA, including splicesomal (WBP4, SFRS1, SFRS2IP, SFRS11, SF3B1, PRPF4) and polyadenylation (CPSF6) proteins (Figure S3B; Supplemental Information S1, worksheet D). Epithelial network 3 included genes encoding molecules that function in the cytoplasm to regulate translation and included 7 eukaryotic translation initiation factors (Figure S3C; Supplemental Information S1, worksheet E). The majority of the proteins encoded by the genes within epithelial network 4 function within the nucleus and included 6 mediator complex genes (MED4, MED9, MED10, MED13L, MED25, MED31), which co-activate DNA-binding factors that activate transcription via RNA polymerase II, and 5 RNA polymerase genes (POLR1E, POLR2B, POLR3B, POLR3F) (Figure S3D; Supplemental Information S1, worksheet F). Eight proteins encoded by genes in epithelial network 5 make up the NADH-ubiquinone oxidoreductase complex, which is part of the mitochondrial respiratory chain and serves to catalyze oxidation of NADH and the reduction of ubiquinone. Other genes in this network encoded several RAB proteins, which facilitate the docking and transport of vesicles, a process important to secretory activity (Figure S3E; Supplemental Information S1, worksheet G).

**Table 3:** Biological functions of genes in the top 5 networks generated in IPA; (scores) and [central molecules] within networks.

Higher in epithelium	Higher in stroma
Protein synthesis, transcription, translation, cytoskeletal (49) [FOS]	Tissue development, extracellular matrix (51) [NID/Laminins]
RNA post-transcriptional modification, cellular assembly and organization, DNA replication, recombination and repair (46) [WBP4, MAPK]	Tissue morphology, inflammation (44) [IL6]
Protein synthesis RNA post-transcriptional modification, gene expression (38)	Cellular assembly and organization, cellular function and maintenance and cell movement (37) [dynamin]
Gene expression, cell movement, cell to cell signaling and interaction (38) [MR124]	Cell-to-cell signaling and interaction, cell-ECM interaction (36) [TGFBR2, integrins, collagens]
Secretory activity, mitochondrial respiration (36) [RIF1]	Cell death/survival, extracellular matrix (36) [FN1]

Molecules in stromal network 1 encoded for basement membrane proteins including laminins, NID1 and NID2, and FBLN1,2 and 5 (Figure S4A; Supplemental Information S1, worksheet H). The primary hub molecule in stromal network 2 was IL-6, which encodes a cytokine that functions in inflammation and the maturation of B cells and may be important to organ development, and its receptor (Figure S4B; Supplemental Information S1, worksheet I). Stromal network 3 had several hubs that encoded molecules involved in clathrin mediated endocytosis (ITSN1, DNM1, AP2M1, AP2S1) (Figure S4C; Supplemental Information S1, worksheet J). Genes within stromal network 4 encoded proteins that mediate cell-ECM connections (ITGA5, ITGB3, BCAM), were indicative of ECM synthesis (collagens, SPARC, DCN) and breakdown (SERPINA5, MMP14) as well as growth factor receptors that regulate these processes (TGFBR2, ENG) (Figure S4D; Supplemental Information S1, worksheet K). FN1 (fibronectin) was the primary hub molecule of stromal network 5. A node in this network connected FN1 with SNAI2. Specifically, the node indicated that FN1 expression was induced by SNAI2 (snail homologue 2), a gene that encodes a protein that regulates cell survival, epithelial-mesenchymal transition, proliferation, and migration (Figure S4E; Supplemental Information S1, worksheet L).

### Molecular and cellular functions and canonical pathways enriched with genes expressed at greater levels in epithelial or stromal compartments

Canonical pathways enriched with genes expressed at a greater level in epithelial or stromal compartments were also examined using IPA. The canonical pathway most highly enriched in the epithelium was the Protein Ubiquitination Pathway (34 genes; P = 0.0001). Other epithelial enriched pathways included Oxidative Phosphorylation (28 genes; P = 0.0004), IGF-I Signaling Pathway (15 genes; P = 0.01) and Neuregulin Signaling (12 genes; P = 0.03; Table 4). Hepatic Fibrosis/Hepatic Stellate Cell Activation (28 genes; P<0.0001) was the canonical pathway most highly enriched in stromal fibroblasts (Table 4). Other pathways enriched in stromal tissue included Dendritic Cell Maturation (16 genes; P = 0.0007), IL6 signaling (10 genes; P = 0.01) and Notch Signaling (6 genes; P = 0.01).

**Table 4:** Canonical pathways enriched with genes expressed higher in epithelial or stromal tissue.

Canonical Pathway	Score*	Ratio†
Epithelial		
Protein ubiquitination pathway	3.7	0.17
Oxidative phosphorylation	3.4	0.16
Clathrin-mediated endocytosis signaling	2.5	0.15
Mitochondrial dysfunction	2.2	0.14
P70S6K signaling	2.1	0.15
PPAR signaling	2.0	0.15
PI3K/AKT signaling	2.0	0.14
Renal Cell carcinoma signaling	1.9	0.18
Angiopoietin signaling	1.9	0.17
Aldosterone signaling in epithelial cells	1.8	0.14
Butonoate metabolism	1.8	0.10
IGF-1 signaling	1.8	0.15
Aryl hydrocarbon receptor signaling	1.7	0.13
LPS-stimulated MAPK signaling	1.6	0.10
Cell cycle: G2/M DNA damage check point regulation	1.5	0.17
14-3-3 mediated signaling	1.5	0.20
Xenobiotic metabolism signaling	1.5	0.15
NRF2-mediated oxidative stress response	1.5	0.10
Citrate cycle	1.5	0.12
PDGF signaling	1.5	0.11
Neuregulin signaling	1.5	0.12
Stromal		
Hepatic fibrosis/hepatic stellate activation	11	0.20
Dendritic cell maturation	3.0	0.08
Complement system	2.6	0.18
Leukocyte extravasation signaling	2.5	0.09
Virus entry via endocytic pathways	2.5	0.10
ILK signaling	2.5	0.12
Caveolar-mediated endocytosis signaling	2.3	0.13
Crosstalk between dendritic cells and natural killer cells	2.2	0.18
TREM1 signaling	2.0	0.10
Acute phase signaling	1.8	0.08
IL-6 signaling	1.8	0.11
NOTCH signaling	1.8	0.12
LXR/RXR Activation	1.7	0.10
Macropinocytosis signaling	1.4	0.10
Clathrin mediated endocytosis	1.4	0.08
Graft versus host disease signaling	1.3	0.10
IL-8 signaling	1.3	0.08
Actin cytoskeleton signaling	1.3	0.06

*score = −log(P-value); ^†^ratio = number of genes enriching set/total number of genes in set.

**Figure 4:**
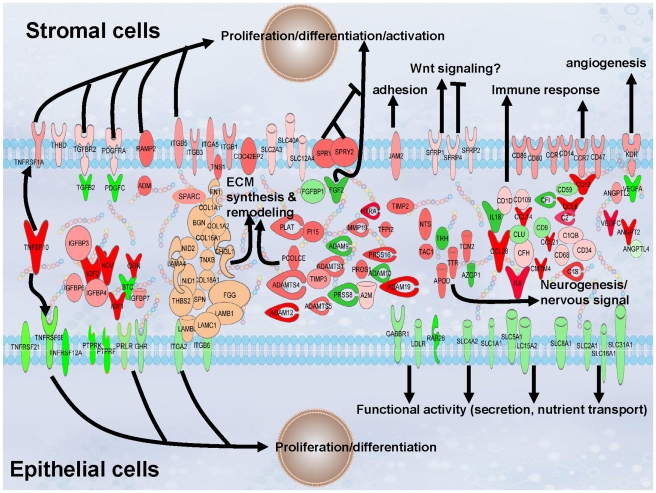
Epithelial (shades of green) and stromal (shades of red) contributions to extracellular and plasma membrane signatures of the mammary transcriptome in three week prepartum dry cows. Gene names, fold differences in expression, P-Value, and FDR are given in Supplemental Information S1, worksheet M.

To investigate whether there was transcriptome evidence for stromal and epithelial interactions within these classical signaling pathways, gene sets that were expressed at a greater level in either of the two tissues at P<0.05 were analyzed using IPA. Three canonical pathways showed evidence of interactions between the cell types: Hepatic Fibrosis/Hepatic Stellate Cell Activation Pathway, the IGF-1 Signaling Pathway and the VEGF Signaling Pathway (Supplemental Information S1, worksheets M, N, and O, respectively). Potential paracrine interactions were also investigated by sorting molecules in IPA within the molecule tab by function and obtaining lists of genes that encode proteins expressed in the plasma membrane or secreted from the cell. These genes were exported into the Pathway Designer tool and the majority were used to generate Figure 4, which illustrates epithelial and stromal fibroblast contributions to the mammary transcriptome during this stage of development (Supplemental Information S1, worksheet P lists all gene symbols, names, fold change, P-value and FDR). Growth factors and receptors (G-protein coupled and transmembrane) differentially expressed between the compartments were also examined to gain insight into tissue interactions and responsiveness (P<0.05; Table 5 and Supplemental Information S1, worksheet Q, respectively). Eight growth factors/cytokines and twenty-two receptors were expressed at a greater level in the epithelial compartment (Supplemental Information S1, worksheet Q). Receptors expressed at a greater level in the epithelium included the prolactin receptor (PRLR), growth hormone receptor (GHR), FGFR2 and 2 claudin receptors (CLDN3, CLDN4). Fifty-two receptors (Supplemental Information S1, worksheet Q) and twenty-one growth factors/cytokines (Table 5) were expressed at a greater level in the intralobular stromal compartment. Receptors expressed at a greater level in the stromal compartment included three secreted frizzled-related proteins, SFRP1, SFRP2, and SRRP4, which are known to mediate Wnt signaling. Expression of these three proteins was 42, 133, and 7-fold greater, respectively, in stroma than epithelium.

**Table 5:** Growth factors expressed at a greater level in epithelial or intralobular stromal cells.

Symbol	Name	Fold change*	P-value	FDR
Epithelial				
IL18	interleukin 18 (interferon-gamma-inducing factor)	−2.586	2.60E-02	0.105
SPP1	secreted phosphoprotein 1	−7.247	7.89E-03	0.048
BDH2	3-hydroxybutyrate dehydrogenase, type 2	−5.039	2.22E-04	0.004
CHI3L1	chitinase 3-like 1 (cartilage glycoprotein-39)	−5.845	8.03E-03	0.049
PLA2G12A	phospholipase A2, group XIIA	−3.235	2.77E-02	0.109
SULF2	sulfatase 2	−3.567	5.00E-02	0.162
BTC	betacellulin	−1.415	6.36E-03	0.042
FGF2	fibroblast growth factor 2 (basic)	−2.172	4.15E-02	0.143
PDGFC	platelet derived growth factor C	−3.792	1.90E-03	0.018
TGFB2	transforming growth factor, beta 2	−2.339	1.70E-02	0.08
VEGFA	vascular endothelial growth factor A	−3.442	1.25E-03	0.013
Stromal				
CCL14	chemokine (C-C motif) ligand 14	7.457	1.95E-05	0.001
CCL2	chemokine (C-C motif) ligand 2	10.067	4.55E-07	0
CCL21	chemokine (C-C motif) ligand 21	3.652	1.86E-03	0.018
CCL8	chemokine (C-C motif) ligand 8	3.742	2.91E-03	0.024
CMTM4	CKLF-like MARVEL transmembrane domain containing 4	3.605	1.90E-02	0.086
CSF1	colony stimulating factor 1 (macrophage)	3.67	6.13E-03	0.041
CXCL13	chemokine (C-X-C motif) ligand 13	3.817	1.26E-04	0.002
CXCL16	chemokine (C-X-C motif) ligand 16	2.804	1.07E-03	0.012
CXCL2	chemokine (C-X-C motif) ligand 2	7.308	4.19E-04	0.006
FAM3C	family with sequence similarity 3, member C	2.55	2.12E-02	0.092
IL6	interleukin 6 (interferon, beta 2)	17.019	6.39E-05	0.001
TNFSF10	tumor necrosis factor (ligand) superfamily, member 10	2.329	1.63E-02	0.078
ANGPT2	angiopoietin 2	8.661	2.60E-05	0.001
IGF1	insulin-like growth factor 1 (somatomedin C)	13.635	2.22E-06	0
IGF2	insulin-like growth factor 2 (somatomedin A)	8.739	6.37E-05	0.001
JAG1	jagged 1 (Alagille syndrome)	3.844	8.17E-04	0.01
KITLG	KIT ligand	3.086	1.56E-04	0.003
NOV	nephroblastoma overexpressed gene	2.27	9.22E-03	0.054
OGN	osteoglycin	28.521	0.00E+00	0
PTN	pleiotrophin	2.125	1.79E-02	0.082
VEGFC	vascular endothelial growth factor C	6.419	3.78E-04	0.005

*Fold change is expressed as the difference between stromal-epithelial, thus the level of PDGFC expression is 3.79-fold greater in epithelial cells relative to intralobular stromal fibroblasts.

### Validation of results with q-PCR and IHC

qPCR was used to measure and validate differential expression of FN1, NID1, IGF1, CDH1, PRLR, and CSN2 between epithelial and stromal tissue. Relative expression of FN1, NID1 and IGF1 was more than 150-fold higher in stroma than epithelial tissue. Thus confirming the much higher stromal expression measured with microarray analysis (41.874, 63.481, and 13.635 log base 2, respectively). Similarly the higher expression of CDH1, PRLR and CSN2 in epithelial tissue measured with microarray analysis was validated using q-PCR, again showing greater than 150-fold expression difference between the tissues.

**Figure 5:**
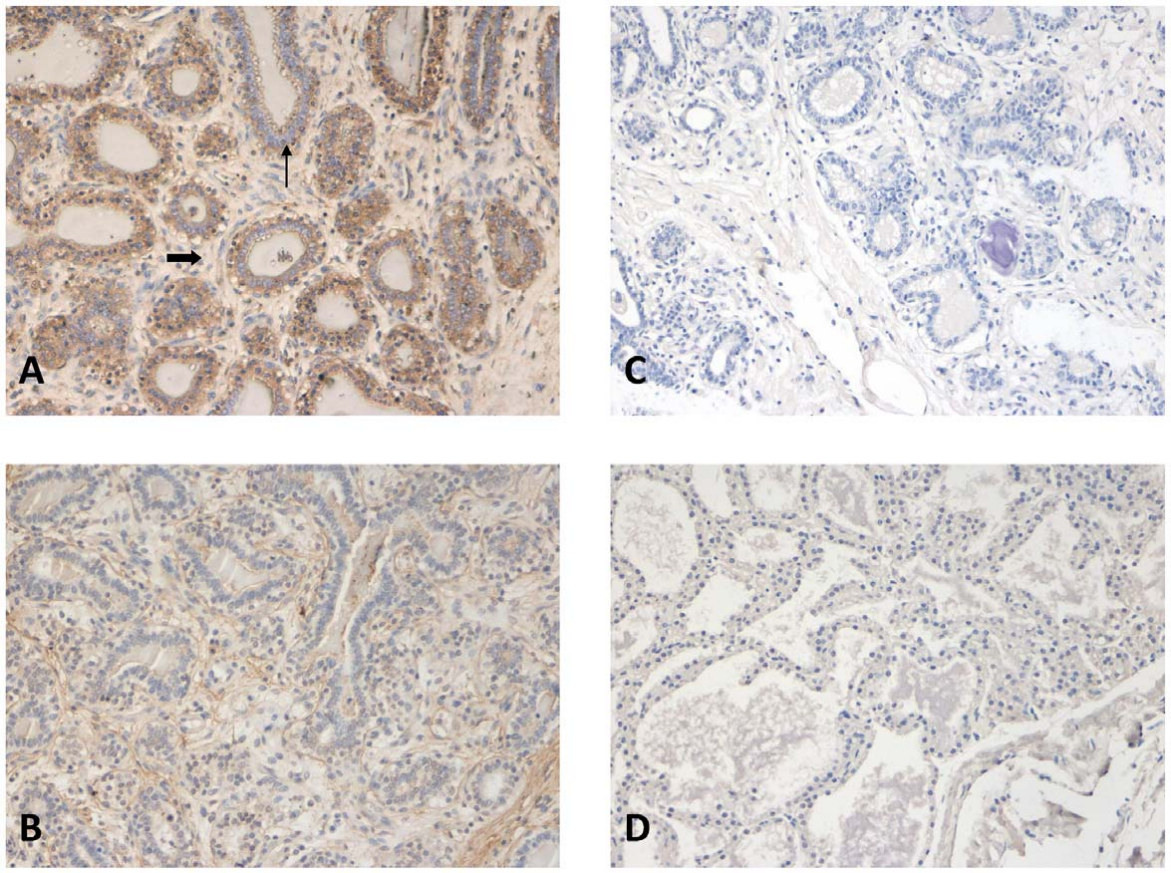
Immunohistochiemical staining shows distribution of fibronectin and MMP3 protein in bovine mammary tissue three weeks prepartum. Images of mammary explant tissue cultured for 2 hrs with insulin and hydrocortisone and captured using light microscopy at 200X following immunohistochemical staining with A) primary mouse polyclonal anti-MMP-3;B) primary mouse monoclonal anti-fibronectin antibody at RT or incubation with diluent alone for C) MMP 3 negative control, and D) fibronectin negative control, followed by incubation with a broad-spectrum secondary antibody conjugated with streptavidin-peroxidase that was visualized with incubation with 3′, 3′-diaminobenzidine chromogen substrate. All slides were counterstained with hematoxylin and cover-slipped before capture.

Distribution of expression of MMP3 and fibronectin proteins that remodel and make up the extracellular matrix, respectively, was investigated using immunohistochemistry (Figure 5). The epithelial compartment exhibited a greater intensity of staining for MMP3 expression than the stromal compartment. MMP3 staining associated with the epithelium was cellular. Stromal MMP3 staining was diffuse and apparently primarily extracellular (Figure 5A). Fibronectin staining was primarily limited to the stromal compartment and showed organization in fibrous sheets (Figure 5B).

## Discussion

Our results demonstrate, for the first time, the unique characteristics of the transcriptomes of epithelial cells and intralobular stromal fibroblasts in mammary tissue of dry cows at three weeks prepartum. Differential gene expression between the compartments was characterized by an enrichment of genes that primarily had intracellular functions in the epithelial compartment and extracellular functions in the stromal compartment. Further, expression analysis enabled the dissecting of the tissue specific contributions for preparing the mammary gland for milk synthesis.

The epithelial transcriptome was enriched with genes that clustered in ontologies and pathways characteristic of cellular turnover and development of protein and lipid synthetic machinery. Specifically, the enrichment of genes in molecular and cellular functions: cellular growth and proliferation, cell death and cell cycle indicate that at this point in mammary development the epithelial compartment is replacing old senescent cells with new cells, and cells are preparing to synthesize proteins and lipids that are necessary for lactation. Enrichment of genes in cellular assembly and organization, RNA trafficking, protein trafficking and energy metabolism molecular and cellular functions is indicative of the development of cellular infrastructure necessary to support the increased rates of protein synthesis and secretory activity needed for lactation [33], [34], [35]. Canonical pathways enriched with genes expressed at a greater level in epithelium reflected its metabolic and synthetic roles (oxidative phosphorylation, citrate cycle, protein ubiquitination pathway) and its role in initiating angiogenesis (angiopoetin signaling) and neurogenesis (neuregulin signaling).

IPA network analysis enabled the visualization of cellular distribution and queries into interactions among molecules encoded by genes that enriched epithelial or stromal transcriptomes. The top five IPA epithelial networks were composed of genes that encoded for molecules with intracellular functions. Functions of these molecules included regulators of transcription, translation and intracellular trafficking, reflective of the role of epithelial cells in the mammary and the building of cellular machinery necessary for milk synthesis [33], [34], [35].

Genes in stromal transcriptome enriched cellular ontologies specific for ECM and secreted molecules. Enriched cellular and molecular functions indicated that genes expressed at a greater level in the intralobular stroma encoded proteins that regulate cell-cell signaling, cellular movement, and cellular development. Stromal molecular signatures also reflected that cell turnover, proliferation and death occurred in mammary stroma of three week prepartum dry cows. Specifically, molecular and cellular functions: cellular growth and proliferation, cell death and cell cycle were enriched with genes expressed at a greater level in stromal fibroblasts.

Translation of proliferation signatures in both tissues was evident in the presence of approximately 0.34% and 0.37% BrdU labeled epithelial and stromal cells, respectively. Relative rate of stromal cell proliferation was similar to rates reported for stromal fibroblasts in dry cows three weeks prepartum [4]. However the relative rate of epithelial cell proliferation was 10- to 20-fold less than rates previously reported. Norgaard et al. reported that 6% and 12% of bovine mammary epithelial cells expressed the proliferation marker Ki67 at 4 weeks and 2 weeks prepartum [36]. Using a protocol similar to ours, Capuco et al. reported an epithelial labeling index of 2.8% in 3 week prepartum dry cows [4]. Further, in the Norgaard study, tissue was immediately fixed after biopsy, while for both Capuco's study and our study tissue was incubated for 2 hrs in culture with a uridine label to attain labeling index prior to fixation. Thus differences in relative rates of epithelial cell proliferation, may be due to differences in approaches used to attain indexes and/or stages sampled. The lower rate in our study and Capuco's study relative to the Norgaard study also suggest that, although tissue integrity was maintained in short term culture (see Figure 5), epithelial cells in tissue culture have a lower rate of proliferation. In this regard, it is important that we also acknowledge that the transcriptional repertoire may be affected by the 2 hrs of explant culture. Our intent was to maintain state of differentiation by incubating tissue with Ins and Hyd [37]. However transition into an *in vitro* environment also removes the progesterone block, which may lead to secretory activation (for review [37]), and thus may have induced elements of the epithelial signature indicative of lipid synthesis and secretion.

The top five stromal networks had a large proportion of genes that encoded extracellular molecules including laminins, known to regulate milk protein synthesis [17], [38], multiple proteases including MMP and ADAM metallopeptidases, which release signaling factors from the ECM, and several growth factors including IL6, IGF1, and NOV. Cytosolic proteins encoded by genes within the stromal network were primarily related to clathrin-mediated endocytosis. Endocytosis is required for a vast number of functions that are essential for the well being of the cell, including regulation of nutrient uptake, cell adhesion and migration, and receptor signaling. Thus enrichment of these genes likely reflected the very active state of stromal tissue during this period of mammary development.

Mammary stroma is a heterogeneous environment consisting of multiple cell types including fibroblasts, immune, endothelial and nerve cells. Several of the canonical pathways enriched with genes expressed at a greater level in the intralobular stromal compartment reflected this heterogeneous environment. These pathways included Dendritic Cell Maturation, Leukocyte Extravasation Signaling, Complement System, VEGF Signaling and Acute Phase Response Signaling. Enrichment of these pathways suggests that stromal compartment regulates these processes. It is unclear whether these signatures were derived from enriched fibroblasts or other stromal cell types that may have been captured during LCM, as genes that enrich these pathways may be transcribed by stromal fibroblasts and act on corresponding tissues in a paracrine manner or may be indicative of the presence of nervous tissue, endothelial cells and/or immune cells in the stromal isolates.

Enrichment of genes expressed at a greater level in intralobular stroma in the Hepatic Fibrosis/Hepatic Stellate Cell Activation canonical pathway, stromal network 3 (collagens, TGFBR2, SPARC, ENG), ECM gene ontology and VEGF signaling pathway suggest that stromal activation is being induced during this period of mammary gland development. Stromal activation is associated with remodeling during wound healing and pathologically with cancer and fibrosis [39] and is likely a critical component of normal mammary remodeling during the prepartum period. Stromal activation is characterized by fibroblast activation to the myofibroblast phenotype, stimulation of collagen type I deposition and induction of angiogenesis [40]. Myofibroblasts express smooth muscle α-actin, produce proteases such as uPA and stromelysin-3 as well as ECM molecules including collagen I, II and III, fibronectin, and tenascin [41], [42]. It is possible that stromal activation is initiated by transforming growth factor beta 2 (TGFB2) and vascular endothelial growth factor (VEGF) secreted by epithelial cells. TGFB activates mammary fibroblasts into myofibroblasts *in vitro* [43] and *in vivo* [44]. We have also shown that TGFB1 increases the number of myofibroblasts in mammary fibroblasts from dry cows, which supports this signature (unpublished data).

There was also transcriptome evidence for paracrine interactions between the tissues with the enrichment of the IGF1 canonical pathway and expression of IGF binding proteins. The paracrine effects of stromally derived IGF1 on epithelial cell proliferation in the bovine mammary gland is well established [45], [46], [47]. Our stromal and epithelial transcriptomes were consistent with these studies and the molecular signatures suggest that the IGF1 signaling pathway leading to proliferation of epithelial cells is stimulated via IGF1 that was transcribed in the stromal compartment.

Examination of genes that encode proteins with plasma membrane and extracellular distributions gave insight into the interaction between the tissues and pathways that may be active during this period of mammogenesis (Figure 4). Epithelial and stromal contributions to angiogenesis, neurogenesis, immune response and tissue remodeling were unique and were reflected in differential expression of several VEGF molecules, cytokines, and protease molecules. The neurogenesis signatures suggest that both stromal and epithelial tissue participate in increasing nervous innervation of the mammary gland prior to lactation. Increased innerveration of the mammary gland during pregnancy likely reflects preparation for the neuroendocrine response to suckling/milking that is important to milk let down and maintaining lactation. Greater expression of GHR and PRLR transcripts in the epithelial compartment suggests the mammary gland was able to respond to the systemic effects of respective hormones during this period of development, although this would need to be confirmed with protein expression. Differences in expression of growth factors and receptors suggested that local signals for cellular proliferation and differentiation were mediated by paracrine interactions between the compartments at this time. For example higher expression of the growth factors that stimulate fibroblast proliferation/activation (TGFB2 and PDGF) in the epithelial compartment was mirrored by higher expression of their receptors (TGFBR2 and PDGFR) in the stromal compartment.

Interestingly although both epithelial and stromal transcriptomes showed differential contributions to the expression of proteases that remodel the ECM, only the stromal compartment showed evidence of expression of ECM molecules. These signatures were validated using immunohistochemistry. Staining showed the differential distribution of proteins that make up the extracellular matrix, fibronectin, and remodel the ECM, MMP3, as well as TGFB1 and its type II receptor (TGFBR2) (Figure 5 and [6]).

Both compartments showed unique expression of integrin receptors that serve as cell-ECM receptors and are responsible for initiating signals important to cell proliferation and differentiation. These findings suggest that stromal fibroblasts play an important role in regulating mammary function and differentiation through the generation of developmentally specific ECM molecules. The greater expression of SFRP (secreted fizzled-related proteins) in the stromal cell compartment also suggested a role for Wnts and related molecules in mediating mammogenesis during this stage of development. SFRPs have been shown to block Canonical Wnt Signaling pathways and alternatively to behave in a paracrine manner to stimulate epithelial branching [48].

### Conclusion

Mammary epithelial and intralobular stromal cells contribute distinct molecular signatures to the mammary transcriptome in dry cows at 3 weeks prepartum. The epithelial signature was characterized by enrichment of molecules that regulate protein synthesis, metabolism and secretion. In contrast the stromal signature was primarily characterized as being enriched with genes that encode extracellular molecules important to signaling, ECM synthesis and ECM remodeling. Tissue specific signatures also provided evidence of paracrine stimulation of the IGF1 signaling pathway in the epithelial compartment from stromally produced IGF1 and stimulation of the stromal reaction from the epithelial compartment. Genes representing angiogenesis and neurogenesis were transcribed in both the epithelium and stroma. Molecular signatures point to the dynamic role the stroma plays in synthesis of signaling molecules that stimulate epithelial morphogenesis and proliferation, as well as in the synthesis of ECM conducive to milk protein synthesis. These signatures also highlight the importance of examining the roles of cells within the mammary gland when targeting therapies and studying mechanisms that impact milk production.

## Supporting Information

Figure S1 **Experimental design flowchart shows the relation of the results reported in this manuscript to the larger study.** The larger study USDA-CSREES-NRI grant *The stromal effect: Remodeling and the effect of transforming growth factor- beta on the mammary gland during the dry period MICL08379.* Yellow boxes highlight treatments and results that were the focus of this report.
(TIF)

Figure S2 **Distribution of genes differentially expressed between epithelial and stromal tissue by P-value.** Y-axis  = frequency (no.) of genes; x-axis =  median P-value.
(TIF)

Figure S3 **IPA generated networks of genes expresses at a greater level in the epithelium (green).** A) Network 1 Protein synthesis, cardiac dysplasia, cardiovascular disease (Score 49), B) Network 2 RNA post-transcriptional modification, cellular assembly and organization, DNA replication, recombination and repair (score 46), C) Network 3 Protein synthesis RNA post-transcriptional modification, gene expression (score 38) D) Network 4 Gene expression, cell movement, cell to cell signaling and interaction (score 38), E) Cell morphology, digestive system development and function, inflammatory response (score 36) . See below for legend.
(TIF)

Figure S4 **IPA generated networks of genes expresses at a greater level in the stroma (red).** A) Network 1 Tissue development, dermatologic disease and conditions, genetic disorders (score 51), B) Network 2 Cancer, tumor morphology, tissue morphology (score 44), C) Network 3 Cellular assembly and organization, cellular function and maintenance and cell movement (score 37) , D) Network 4 Cardiovascular system development and function, cell-to-cell signaling and interaction, connective tissue disorders (score 36), E) Network 5 Cell death, connective tissue disorders, dermatologic disease and conditions (score 36).
(TIF)

Supplemental Information S1 **Worskeets A–Q.** Lists the category of gene set (e.g. CC =  cellular location; BP =  Biological process; MF = molecular function); term (i.e. specific gene ontology (GO) with GO number); count (no. of genes enriching term); % (percent of total of genes that belong to category enriched by analyzed gene set); P-value (i.e. enrichment of gene set); genes (list of genes enriching gene set by Affymetrix ID); Bonferroni; Benjamini and FDR (false discovery rate) for functional annotation clustering of genes expressed at a greater level in A) epithelium and B) stromal tissue. Symbol, Entrez Gene Name, Affymetrix ID, Fold Change, P-value and False Discovery Rate are listed for C) Genes in IPA generated epithelial network 1; D) Genes in IPA generated epithelial network 2; E) Genes in IPA generated epithelial network 3; F) Genes in IPA generated epithelial network 4; G) Genes in IPA generated epithelial network 5; H) Genes in IPA generated stromal network 1; I) Genes in IPA generated stromal network 2; J) Genes in IPA generated stromal network 3; K) Genes in IPA generated stromal network 4; L) Genes in IPA generated stromal network 5; M) Gene enrichment of Hepatic Fibrosis/Hepatic Stellate Cell Activation Canonical Pathway; N) Genes clustered in IGF canonical pathway; O) Gene enrichment of VEGF signaling canonical pathway. P) Genes in Figure 4; Q) Receptors expressed at a greater level in epithelial or stromal tissue in three week prepartum dry cows.
(XLS)
