# Supplementary figures and images for: Transcriptome Analysis of Epithelial and Stromal Contributions to Mammogenesis in Three Week Prepartum Cows
Source: PLoS One. 2011 Jul 29;6(7):e22541. doi: 10.1371/journal.pone.0022541 (PMC3146472; doi:10.1371/journal.pone.0022541)

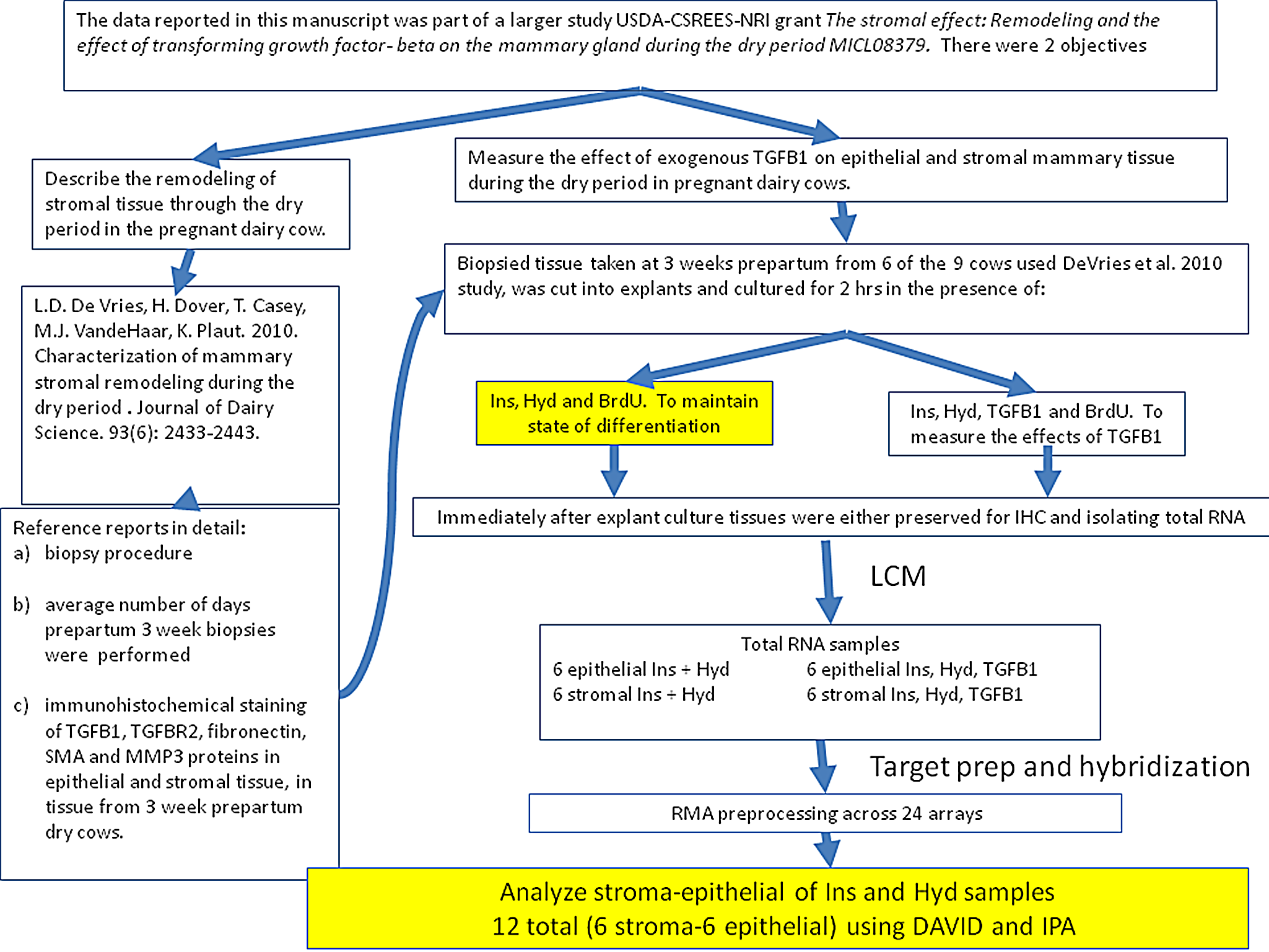

Supplement: Figure S1 — Experimental design flowchart shows the relation of the results reported in this manuscript to the larger study. The larger study USDA-CSREES-NRI grant The stromal effect: Remodeling and the effect of transforming growth factor- beta on the mammary gland during the dry period MICL08379. Yellow boxes highlight treatments and results that were the focus of this report. (TIF) [file pone.0022541.s001.tif]

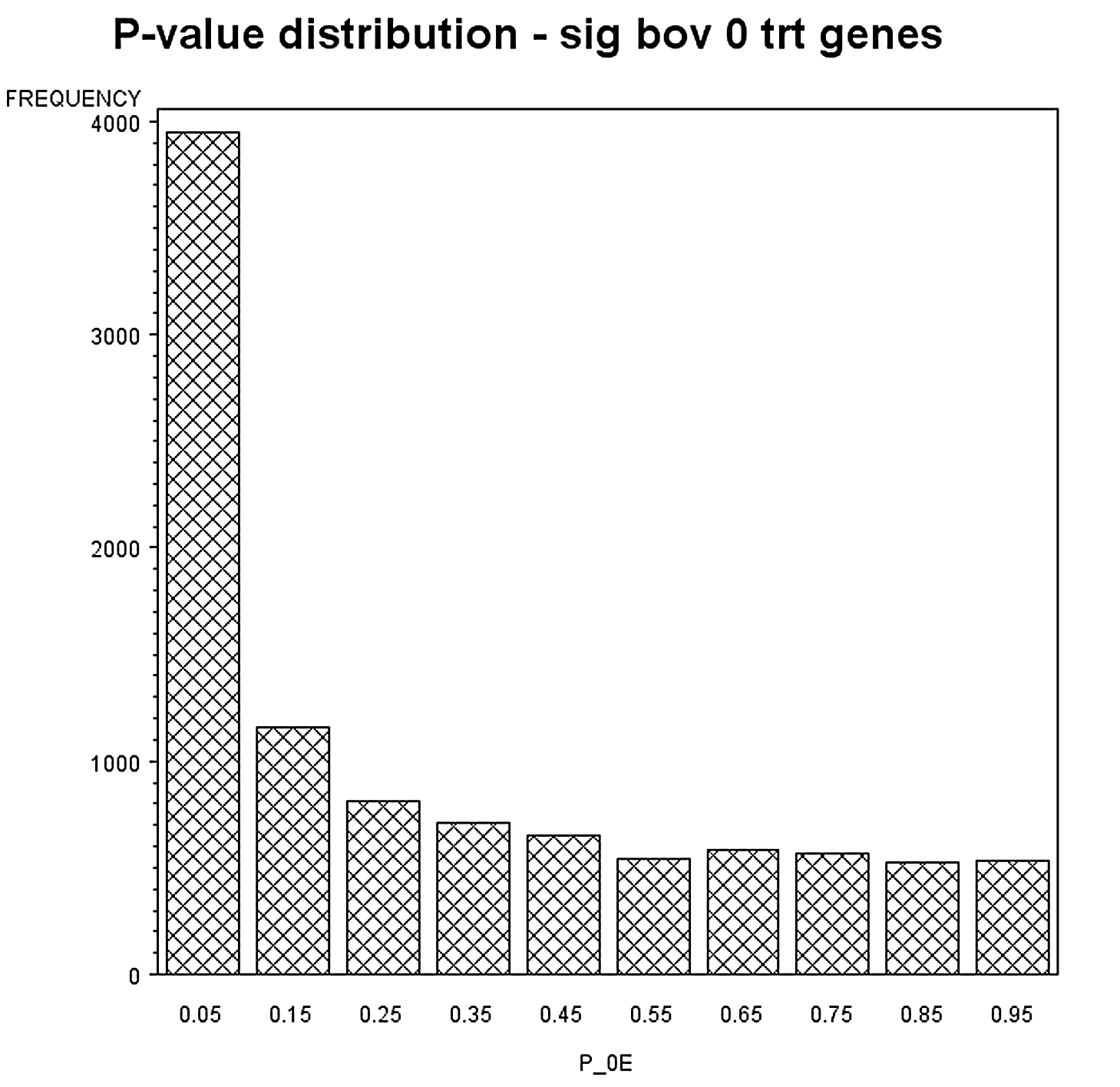

Supplement: Figure S2 — Distribution of genes differentially expressed between epithelial and stromal tissue by P-value. Y-axis = frequency (no.) of genes; x-axis = median P-value. (TIF) [file pone.0022541.s002.tif]

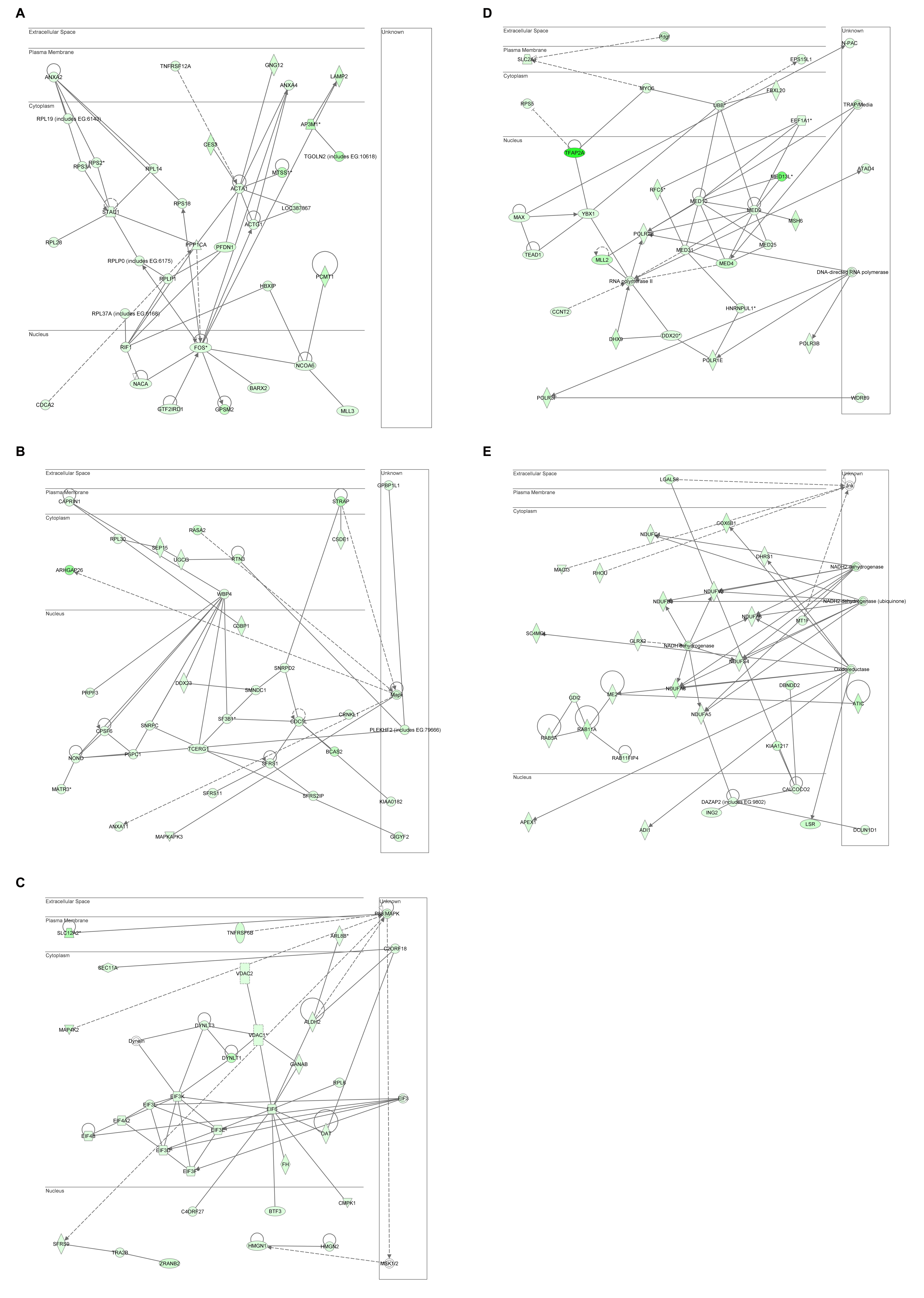

Supplement: Figure S3 — IPA generated networks of genes expresses at a greater level in the epithelium (green). A) Network 1 Protein synthesis, cardiac dysplasia, cardiovascular disease (Score 49), B) Network 2 RNA post-transcriptional modification, cellular assembly and organization, DNA replication, recombination and repair (score 46), C) Network 3 Protein synthesis RNA post-transcriptional modification, gene expression (score 38) D) Network 4 Gene expression, cell movement, cell to cell signaling and interaction (score 38), E) Cell morphology, digestive system development and function, inflammatory response (score 36) . See below for legend. (TIF) [file pone.0022541.s003.tif]

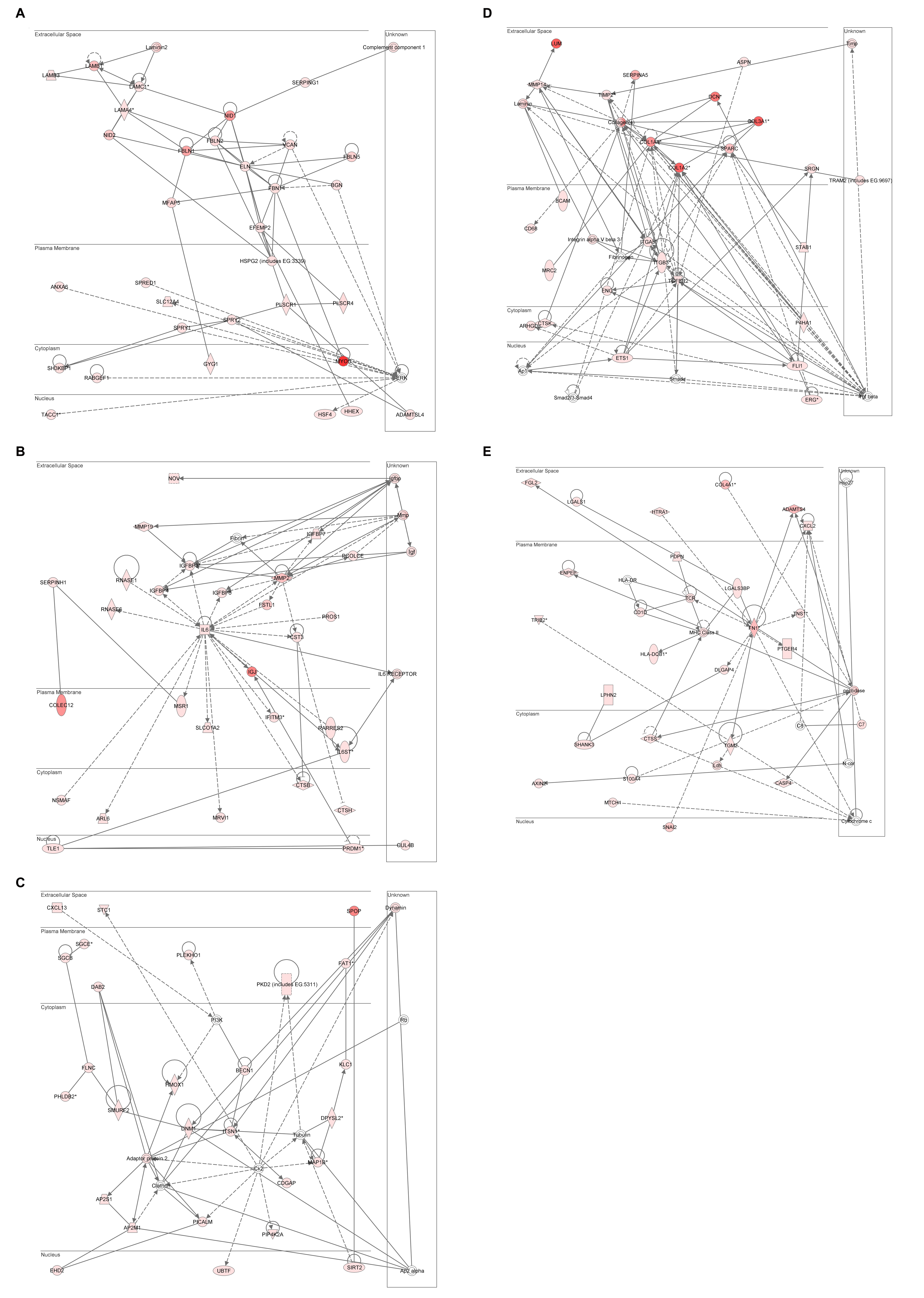

Supplement: Figure S4 — IPA generated networks of genes expresses at a greater level in the stroma (red). A) Network 1 Tissue development, dermatologic disease and conditions, genetic disorders (score 51), B) Network 2 Cancer, tumor morphology, tissue morphology (score 44), C) Network 3 Cellular assembly and organization, cellular function and maintenance and cell movement (score 37) , D) Network 4 Cardiovascular system development and function, cell-to-cell signaling and interaction, connective tissue disorders (score 36), E) Network 5 Cell death, connective tissue disorders, dermatologic disease and conditions (score 36). (TIF) [file pone.0022541.s004.tif]
